# Comparison of hemolytic activity of the intermediate subunit of *Entamoeba histolytica* and *Entamoeba dispar* lectins

**DOI:** 10.1371/journal.pone.0181864

**Published:** 2017-07-27

**Authors:** Kentaro Kato, Takashi Makiuchi, Xunjia Cheng, Hiroshi Tachibana

**Affiliations:** 1 Department of Eco-epidemiology, Institute of Tropical Medicine (NEKKEN), Nagasaki University, Nagasaki, Japan; 2 Department of Infectious Diseases, Tokai University School of Medicine, Isehara, Kanagawa, Japan; 3 Department of Medical Microbiology and Parasitology, School of Basic Medical Sciences, Fudan University, Shanghai, China; Universita degli Studi di Parma, ITALY

## Abstract

Galactose and *N*-acetyl-D-galactosamine-inhibitable lectin of *Entamoeba histolytica* has roles in pathogenicity and induction of protective immunity in rodent models of amoebiasis. Recently, the intermediate subunit of the lectin, Igl1, of *E*. *histolytica* has been shown to have hemolytic activity. However, the corresponding lectin is also expressed in a non-virulent species, *Entamoeba dispar*, and another subunit, Igl2, is expressed in the protozoa. Therefore, in this study, we compared the activities of Igl1 and Igl2 subunits from *E*. *histolytica* and *E*. *dispar* using various regions of recombinant Igl proteins expressed in *Escherichia coli*. The recombinant *E*. *dispar* Igl proteins had comparable hemolytic activities with those of *E*. *histolytica* Igl proteins. Furthermore, *Igl1* gene-silenced *E*. *histolytica* trophozoites showed less hemolytic activity compared with vector-transfected trophozoites, indicating that the expression level of Igl1 protein influences the activity. These results suggest that the lower hemolytic activity in *E*. *dispar* compared with *E*. *histolytica* reflects the lower expression level of Igl1 in the *E*. *dispar* parasite.

## Introduction

Amoebiasis due to infection with *Entamoeba histolytica* (*E*. *histolytica*) is a problematic parasitic disease in many countries. *E*. *histolytica* causes an estimated 50 million cases of dysentery, colitis and extraintestinal abscesses, resulting in 40,000 to 100,000 deaths annually [1]. Adherence of *E*. *histolytica* trophozoites to colonic mucins and host cells is mediated by a galactose (Gal)- and *N*-acetyl-D-galactosamine (GalNAc)-inhibitable lectin [2]. This lectin is a 260-kDa heterodimer of two glycoproteins: a transmembrane heavy subunit (Hgl, 170 kDa), one of the key molecules in amebic adherence, and a glycosylphosphatidylinositol (GPI)-anchored light subunit (Lgl, 35/31 kDa).

Another GPI-anchored 150-kDa intermediate subunit (Igl, 150 kDa) is non-covalently associated with the Hgl/Lgl dimer in different lipid raft-like domains and also contributes to adherence [3–5]. There are two isoforms of Igl, which are referred to as Igl1 and Igl2, and both contain multiple CXXC motifs with different localization in *E*. *histolytica* trophozoites [6, 7]. These two Igls are also found in *Entamoeba dispar* (*E*. *dispar*), which is morphologically indistinguishable from *E*. *histolytica*, but is non-pathogenic [8]. The expression level of *Igl1* is about twice as high in *E*. *histolytica* HM-1:IMSS than in *E*. *dispar* SAW1734RclAR, whereas that of *Igl2* is comparable in the two species, suggesting that Igl1 may be associated with the pathogenicity of *E*. *histolytica* [8]. In fact, Igl1 is recognized by sera from patients with amoebiasis and is also a vaccine candidate [9, 10].

Igl is a parasitic lectin that binds to *p*-aminophenyl-β-D-galactopyranoside-Sepharose gel in a Gal-affinity column [5]. *E*. *histolytica* Igl has also been detected, in addition to Hgl and Lgl, in the protein fraction that binds to GalNAc bovine serum albumin-coated magnetic beads [11]. Recently, while exploring the lectin domain of Igl, we found that Igl1 of *E*. *histolytica* possesses both hemolytic and cytotoxic activities [12]. However, it is unclear whether Igl1 of *E*. *dispar* and Igl2 of both species have the same activity. Therefore, in this study, we compared the hemolytic activities of *E*. *dispar* Igls with those of *E*. *histolytica* Igls *in vitro*. We also attenuated expression of Igl1 in *E*. *histolytica* utilizing a gene-silencing technique and evaluated the effect on hemolytic activity, since *E*. *dispar* has lower expression of Igl1 compared with *E*. *histolytica* [8].

## Materials and methods

### Expression and refolding of recombinant Igl proteins and Ni column purification of the proteins

Recombinant EhF-Igls, EdF-Igls, EhNM-Igl1, EhM-Igl1, EhC-Igl1 or EdC-Igl1 proteins with a His-tag at the N-terminus were expressed in *Escherichia coli* BL21 Star(DE3)pLysS cells (Invitrogen) or ECOS^™^ competent BL21(DE3) cells (Nippon Gene Co.), using the primers shown in Table 1. The proteins were further purified using a Ni column, as described in detail previously [9, 12].

**Table 1:** Oligonucleotide primers used in the study.

Primer	Position^a^	Sequence (5′ to 3′)^b^	Ref.
(for Eh Igls)			
EhIgl-S14	40–59	CCCTCGAGGATTATACTGCTGATAAGCT	[9, 12]
EhIgl2-S14	40–70	CCCTCGAGGATTATACTGCTGATAAACTCATTAATAACC	[7]
EhIgl-S294	880–898	CCCTCGAGACAGAAGAAAATAAATGTA	[9, 12]
EhIgl-S603	1807–1827	CCCTCGAGGAAGGACCAAATGCAGAAGAT	[9, 12]
EhIgl-AS753	2244–2259	CCCTCGAGTTATAGCCTTTGTTCAGTG	[9, 12]
EhIgl-AS1088^c^	3247–3264	CCCTCGAGTTAAATGCCTTTAGCTCCATT	[9, 12]
(for Ed Igls)			
EdIgl-S14	40–59	CCCTCGAGGAGTACAAAGCTGATAAACT	[8]
EdIgl2-S14	40–63	CCCTCGAGGATTACAAAGCTGATAAACTCATC	[8]
EdIgl-S604	1810–1830	CCCTCGAGGAAGGACCAAATGAAGAAGAT	*
EdIgl-AS1097^c^	3274–3291	CCCTCGAGTTAAATTCCTTTACTTCCATT	[8]

^a^ Nucleic acid numbering is based on the *E*. *histolytica Igl1* and *Igl2* gene sequences (AF337950 and XM_647302) and *E*. *dispar Igl1* and *Igl2* gene sequences (AB287423 and AB287424).

^b^ Nucleotides added for cloning and translation termination are underlined.

^c^ EhIgl-AS1088 and EdIgl-AS1097 are common for EhIgl2 and EdIgl2 respectively.

* This study.

### SDS-PAGE and coomassie brilliant blue staining of purified recombinant proteins

Recombinant proteins (1 μg each) were mixed with SDS sample buffer (Invitrogen) and subjected to SDS-PAGE. The gel was treated with SimplyBlue Safe stain solution (Invitrogen) and incubated until blue bands appeared on the gel [12].

### Hemolytic assays using recombinant lectins and measurement of released hemoglobin

Hemolytic assays and quantification of hemolytic activity were conducted as previously described [12]. Briefly, recombinant Igls (2 μM each, 50 μl) were mixed with 50 μl of horse red blood cell (HoRBC) solution at room temperature and images were taken at several time points. A Hemoglobin B Test Kit (Wako, Osaka, Japan) was used to measure the concentration of hemoglobin in supernatants of RBCs incubated with recombinant proteins or trophozoites for 8 h or 1 h. The results are expressed as the mean of 5 experiments with SD.

### Culturing Entamoeba trophozoites

Trophozoites of *E*. *histolytica* HM-1:IMSS G3 [13] strain were cultivated axenically in Diamond BI-S-33 medium [14] and used for generating *Igl* gene-silenced trophozoites. Trophozoites of *E*. *dispar* SAW1734RclAR strain were grown monoxenically with *Pseudomonas aeruginosa* or with *Crithidia fasciculata* and trophozoites of *E*. *dispar* CYNO9:TPC strain were axenically cultured in YIGADHA-S medium [15].

### Preparation of Igl1 gene-silenced *Entamoeba histolytica* trophozoites

Isolation of total RNA and mRNA from trophozoites and cDNA synthesis were performed as previously described [16]. For silencing of the *Igl1* gene, the DNA fragment from 156- to 408-nt (gs*Igl1*A strain) or from 1- to 466-nt (gs*Igl1*B strain) in *Igl1* was PCR-amplified from cDNA using Phusion DNA polymerase (New England Biolabs) and specific primer sets (gs*Igl1*A strain: sense, 5′-CGA GGC CTC ACT GGA AAT AAT AAG ACA TG-3′; antisense, 5′-GTC GGA GCT CAC CAT CAA CAG TAG TAG ACA TC-3′, gs*Igl1*B strain: sense, 5′-CGA GGC CTC ATG TTT ATT CTT CTT TTA TTC ATA TC-3′; antisense, 5′-GTC GGA GCT CGA CCA ACA CAA TTT TCT GCA TG-3′, containing *Stu* I and *Sac* I recognition sites, respectively). The fragments were digested with *Stu* I and *Sac* I and ligated into a *Stu* I/*Sac* I double-digested psAP-2-Gunma plasmid [17] using a Ligation-Convenience Kit (Nippon Gene Co., Tokyo, Japan). Lipofection of trophozoites and selection and maintenance of transformants were performed as previously described [18].

### Real-time PCR analysis

Real-time PCR was essentially performed as previously described [8]. Briefly, total RNAs of *E*. *histolytica* and *E*. *dispar* trophozoites isolated using a RNeasy Plus Mini Kit (Qiagen) were used for cDNA synthesis with an ExScript^™^ RT Reagent Kit (Takara). Reaction mixtures for quantitative real-time PCR analysis were prepared using SYBR Premix Ex *Taq* II (Takara), specific primers, Rox dye, and the cDNAs. Forty cycles of amplification with recording of fluorescence intensity in each cycle were performed using StepOnePlus^™^ Real-Time PCR System (ABI). Expression levels of *Igl* genes were analyzed using the comparative C_T_ method with *actin* as an internal standard. The experiments were repeated 3 times, including the steps of culture and isolation of RNA.

### Antibodies

Purified human mAb XEhI-20 (anti-EhIgl1) and XEhI-B5 (anti-EhIgl2) were used for detection of Igls of *E*. *histolytica* [7]. Mouse mAb ED2-495 against recombinant Igl2 of *E*. *dispar* was obtained as described previously [8] and characterized before use. Mouse mAb ED2-1 specific for Igl2 of *E*. *dispar* was used as a control [8]. Pooled ascites rich in mouse mAbs were used in the study.

### Dot blot analysis

Recombinant Igls (300 ng) were blotted on a nitrocellulose membrane and then air-dried. Filter strips were blocked with 3% bovine serum albumin in PBS and reacted with ED2-1 or ED2-495 for 30 min. After washing with PBS containing 0.05% Tween-20 (PBST), the strips were incubated with horseradish peroxidase (HRP)-labeled goat anti-mouse IgG antibody (MP Biomedicals) for 30 min. The strips were then washed with PBST and developed with a Konica Immunostaining HRP-1000 kit.

### Immunoblot assay

Whole cell lysates (15 or 20 μg protein/well) were applied to a 5–20% gradient polyacrylamide gel (Atto Corp., Tokyo, Japan) and SDS-PAGE was conducted under reducing or non-reducing conditions, respectively. The proteins in the gel were transferred onto an Amersham^™^ Hybond^™^ P 0.45 PVDF membrane (GE Healthcare) that was then incubated with 5% skim milk in PBST for blocking. Mouse ascites (ED2-495) against *E*. *histolytica* Igls was diluted 500 times with PBST containing 5% skim milk. Rabbit antiserum against ATP sulfurylase (AS) of *E*. *histolytica* was prepared [16] and diluted 500-fold with 5% skim milk in PBST. Anti-mouse and anti-rabbit immunoglobulin F(ab’)2 fragments conjugated with HRP (Amersham) were diluted 3000 times with PBST and used as the secondary antibody. Immunoblot assays with XEhI-20 or XEhI-B5 [7] were performed using SDS-PAGE in a 5–20% gradient gel under non-reducing conditions, with 30 μg of XEhI-20 or XEhI-B5 used as the primary antibody and HRP-labeled goat anti-human IgG antibody (MP Biomedicals) diluted 1000 times with PBST as the secondary antibody. Immobilon^™^ Western (Millipore) was used as a substrate for visualization of the proteins. Detection of chemiluminescence and quantification of band intensities were performed by Ez-Capture MG and CS Analyzer ver. 3.0 (Atto Corp.), respectively.

### Immunofluorescence assay

Sample preparation for IFA was performed as previously described [18, 19]. Briefly, after amoeba transformants were incubated on 5-mm round wells on glass slides, the cells were fixed with 4% paraformaldehyde in PBS for 10 min, washed four times with PBS, and permeabilized with 0.05% Triton X-100 in PBS for 5 min. After blocking with 3% bovine serum albumin in PBS, samples were reacted with ED2-495 (mouse IgG), XEhI-20 (human IgG), or XEhI-B5 (human IgG) diluted 1:50 in PBS and subsequently reacted with secondary antibody diluted 1:500 (Alexa Fluor^®^ 488 goat anti-mouse or human IgG; Life Technologies) in PBS. Fluorescence images were obtained using a LSM510 Meta confocal Microscope (Zeiss) in lambda emission fingerprinting mode [20, 21].

### Hemolytic assay using *Entamoeba histolytica* and *Entamoeba dispar* trophozoites

The assay was conducted as previously described [12, 22] with slight modifications. Briefly, vector-transfected (control) or *Igl1* gene-silenced (gs*Igl1*A or gs*Igl1*B) *E*. *histolytica* or wild-type *E*. *dispar* (SAW1734RclAR strain or CYNO9:TPC strain) trophozoites prepared as described above were harvested and washed with PBS. Then 1×10^5^ trophozoites were mixed with 1% HoRBCs in 100 μl PBS and incubated at 37°C for 1 h. The cell suspension was sedimented at 2000 rpm for 5 min and the concentration of hemoglobin in the supernatant was determined as described above.

### Statistical analysis

Multiple comparisons were performed by ANOVA with a Dunn test, with P < 0.05 considered significant.

## Results

### Recombinant Igls

**Fig 1:**
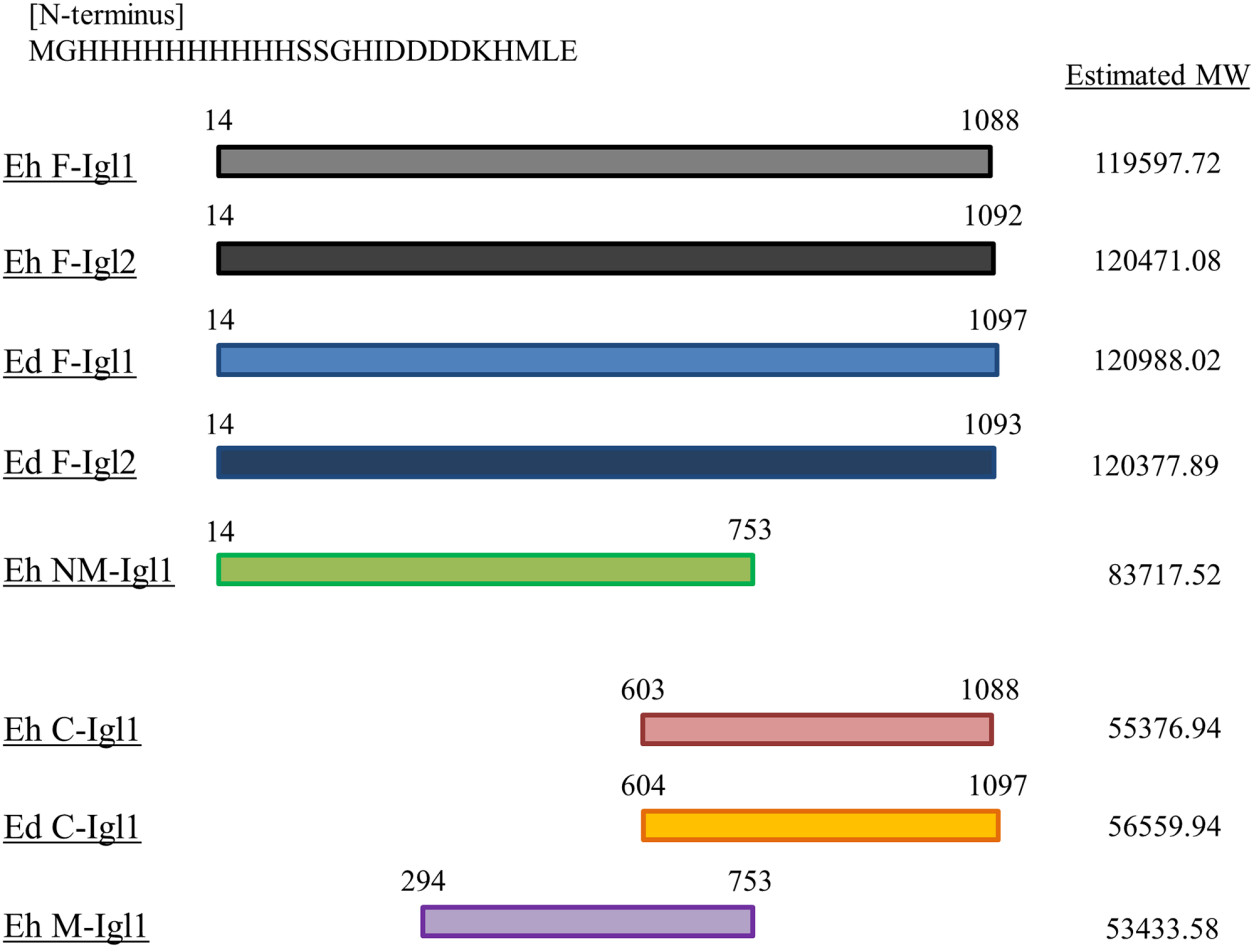
Recombinant Igl proteins used in the study. Recombinant Igl proteins were constructed with a His-tag at the N-terminus. Full length (F-Igl), N-terminus and middle (NM-Igl), middle (M-Igl), and C-terminus (C-Igl) Igl1 and Igl2 of *Entamoeba histolytica* (Eh) and *Entamoeba dispar* (Ed) were expressed in *E*. *coli* and purified using Ni columns. Estimated molecular weights of each protein including the His-tag are shown [ExPASy Compute pI/Mw tool (http://web.expasy.org/compute_pi/)].

**Fig 2:**
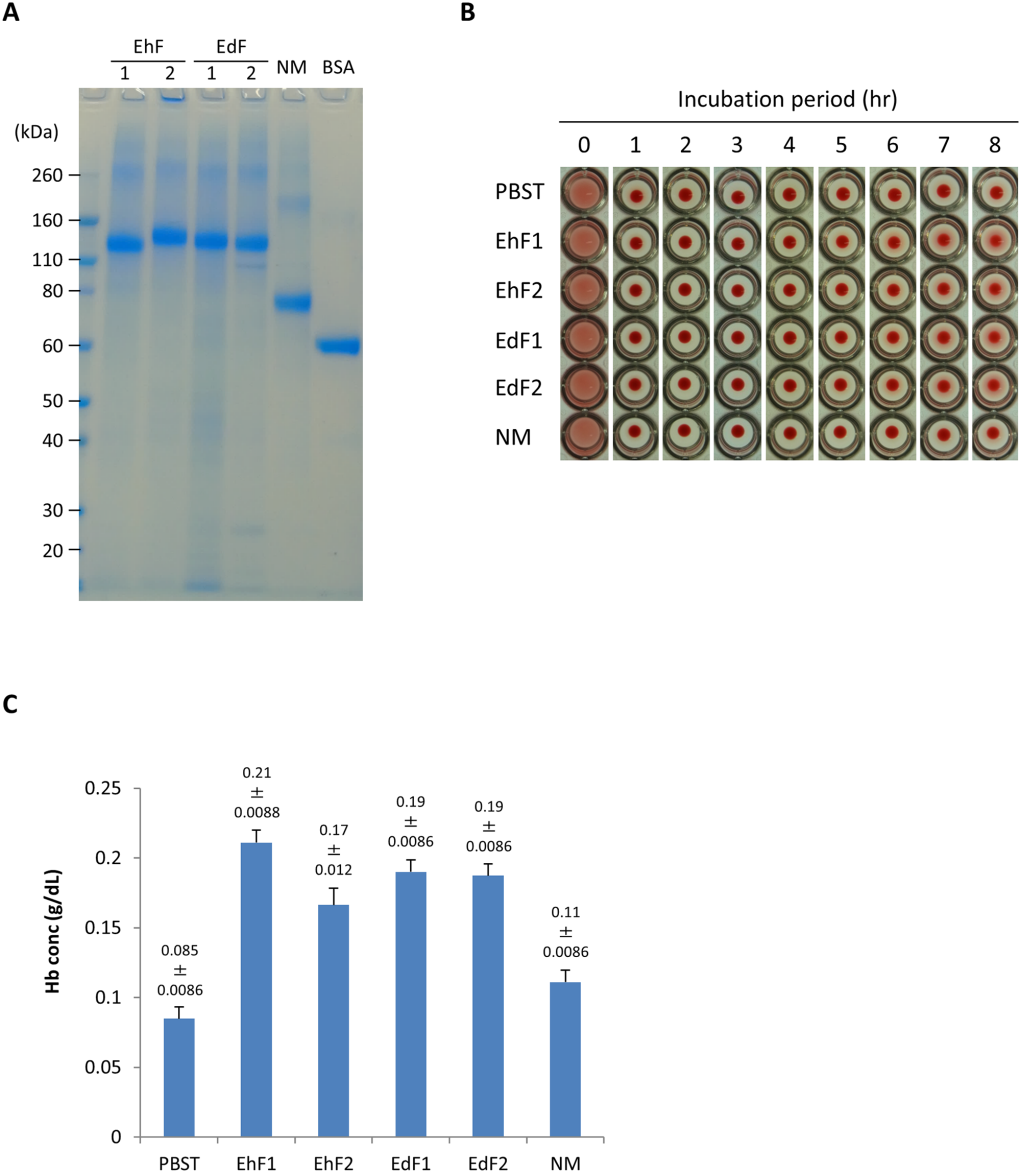
Time-course of hemolytic activities of EhF-Igl1, EhF-Igl2, EdF-Igl1, EdF-Igl2 and EhNM-Igl1 proteins. Recombinant Igl proteins (2 μM, 50 μl) were incubated with 50 μl of 2% (v/v) HoRBCs in PBS for the indicated periods. A. Protein purity and amount were confirmed by SDS-PAGE using NuPAGE Novex Bis-Tris (4–12% gradient) gels with 1 μg of each protein. B. HoRBCs were incubated in a 96-well plate after the indicated periods with Igls. Representative images of 5 independent studies are shown. C. Concentrations of hemoglobin (Hb) released in the supernatant of samples incubated for 8 h. Data are the mean ± SD from 5 independent experiments. EhF-Igls and EdF-Igls showed significantly (**p< 0.01 by ANOVA with Dunn test) higher hemolytic activities than EhNM-Igl1 and PBST. EhF1: EhF-Igl1, EhF2: EhF-Igl2, EdF1: EdF-Igl1, EdF2: EdF-Igl2, NM: EhNM-Igl1.

**Fig 3:**
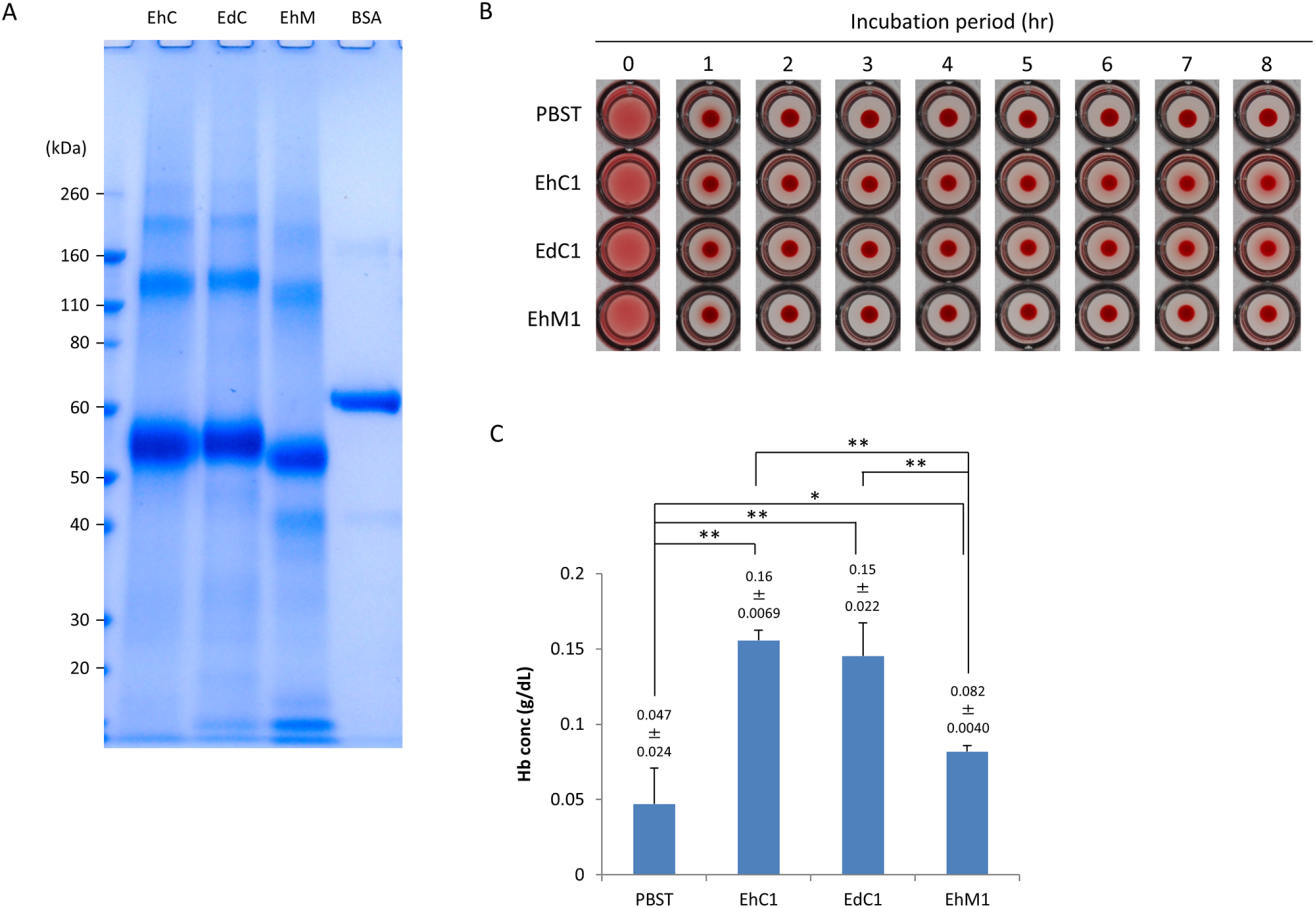
Time-course of hemolytic activities of EhC-Igl1, EdC-Igl1 and EhM-Igl1 proteins. Recombinant Igl proteins (2 μM, 50 μl) were incubated with 50 μl of 2% (v/v) HoRBCs in PBS for the indicated periods. A. Protein purity and amount were confirmed by SDS-PAGE using NuPAGE Novex Bis-Tris (4–12% gradient) gels with 1 μg of each protein. B. HoRBCs were incubated in a 96-well plate after the indicated periods with Igls. Representative images of 5 independent studies are shown. C. Concentrations of hemoglobin (Hb) released in the supernatant of samples incubated for 8 h. Data are the mean ± SD from 5 independent experiments. *p< 0.05, **p< 0.01 by ANOVA with Dunn test. EhC1: EhC-Igl1, EdC1: EdC-Igl1, EhM1: EhM-Igl1.

Full-length (EhF-Igl1: aa 14 to 1088 of *E*. *histolytica* Igl1, EhF-Igl2: aa 14 to 1092 of *E*. *histolytica* Igl2, EdF-Igl1: aa 14 to 1097 of *E*. *dispar* Igl1, EdF-Igl2: aa 14 to 1093 of *E*. *dispar* Igl2), N-terminal and middle (NM-Igl: aa 14 to 753 of *E*. *histolytica* Igl1), middle (M-Igl: aa 294 to 753 of *E*. *histolytica* Igl1), and C-terminal (C-Igl: aa 603 to 1088 of *E*. *histolytica* Igl1 and aa 604 to 1097 of *E*. *dispar* Igl1) regions of *E*. *histolytica* (Eh) and *E*. *dispar* (Ed) Igls with a His-tag at the N-terminus (Fig 1) were expressed in *E*. *coli* [12]. Recombinant proteins were purified using Ni columns, the buffer was changed to PBST, and purities were confirmed by SDS-PAGE (Figs 2A and 3A). The recombinant proteins were then used in further studies.

### Hemolytic activities of recombinant proteins against horse red blood cells (HoRBCs)

We recently showed that EhF-Igl1 has hemolytic activity [12]. EhF-Igl1 and EhF-Igl2 have 83–84% amino acid sequence identity. EdF-Igl1 has 75–76% amino acid sequence identity with EhF-Igl1, and EdF-Igl2 has 73–74% amino acid sequence identity with EhF-Igl2 [8]. To evaluate whether EhF-Igl2, EdF-Igl1 and EdF-Igl2 also have hemolytic activity, HoRBCs (2% v/v) in PBS were mixed with EhF-Igl1, EhF-Igl2, EdF-Igl1, EdF-Igl2 or EhNM-Igl1 (Fig 2B). EhNM-Igl1 was used as a low activity control because it has less hemolytic activity than EhC-Igl1, but a similar molecular weight to EhF-Igls and EdF-Igls [12]. Samples were mixed in U-bottom 96-well plates and incubated at room temperature for up to 8 h to evaluate the hemolytic activities (Fig 2B) based on the concentration of released hemoglobin [Hb] in the supernatant after 8 h (Fig 2C). EhF-Igls and EdF-Igls had significantly higher hemolytic activities than PBST ([Hb] 0.085±0.0086 g dL^-1^) and EhNM-Igl1 (0.11±0.0086 g dL^-1^).

The hemolytic activity of EhF-Igl1 resides in the C-terminus of the protein [12]. To assess whether the C-terminus of EdF-Igl1 also has this activity, we conducted an assay of EhC-Igl1 and EdC-Igl1 (Fig 3), using EhM-Igl1 as a weakly active control. EdC-Igl1 (0.15±0.022 g dL^-1^) and EhC-Igl1 (0.16±0.0069 g dL^-1^) had equivalent activity (Fig 3C), and EhM-Igl1 showed slightly higher activity (0.082±0.0040 g dL^-1^) than PBST (0.047±0.024 g dL^-1^) in an ANOVA test. These results show that EdF-Igl1 has similar hemolytic activity to that of EhF-Igl1 and that the C-terminus of EdF-Igl1 has a role in this activity.

### Hemolytic activities of *Entamoeba dispar* strains

**Fig 4:**
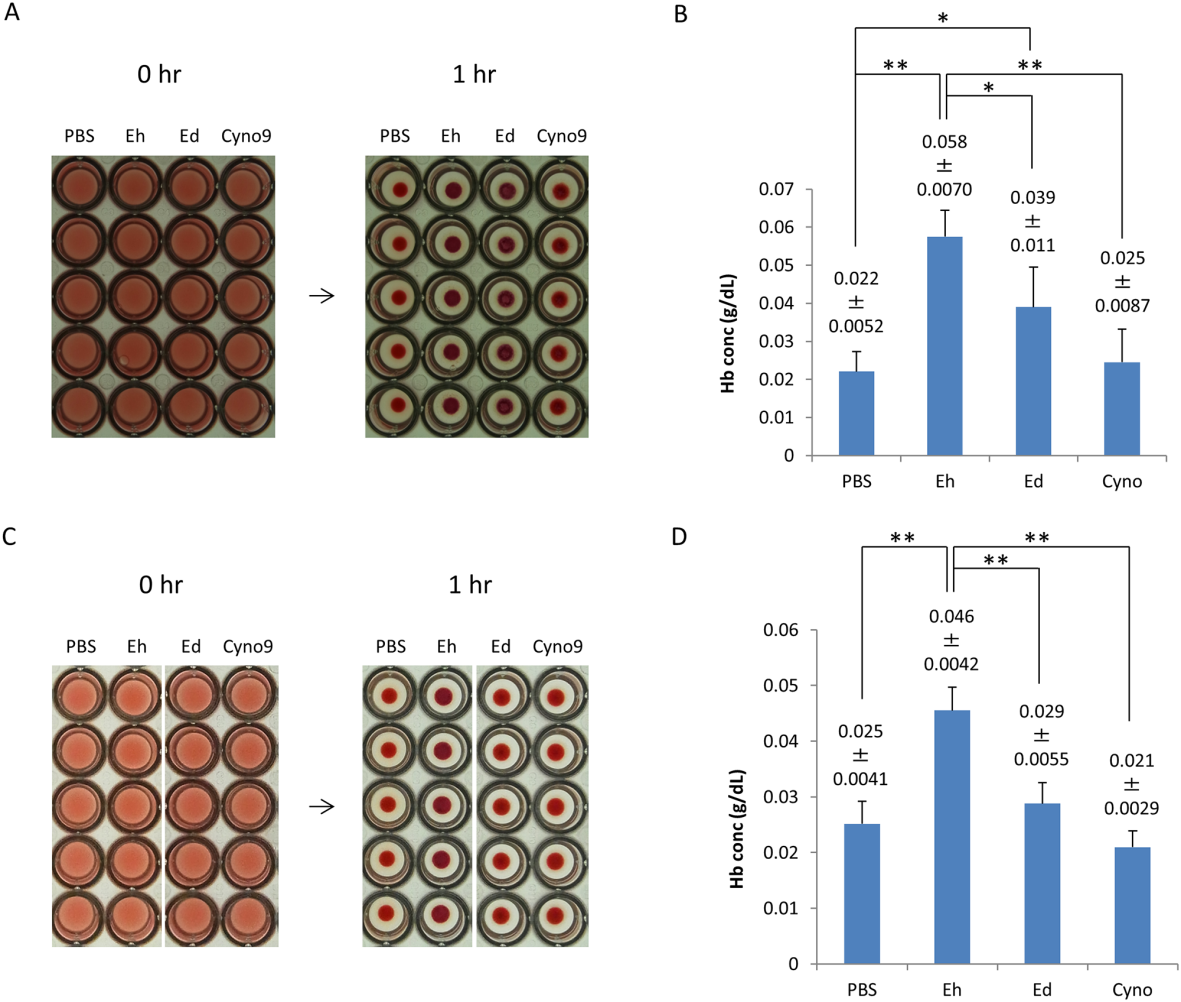
Hemolytic activities of trophozoites of *Entamoeba dispar* strains. A and B. *E*. *histolytica* (Eh), *E*. *dispar* SAW1734RclAR cultured with *Pseudomonas aeruginosa* (Ed) or *E*. *dispar* CYNO9:TPC (Cyno9) trophozoites (1×10^5^) were incubated with HoRBCs at 37°C for 1 h. A. Images of HoRBCs in a 96-well plate just after mixing with trophozoites (0 hr) and after incubation for 1 h with trophozoites (1 hr). B. Released hemoglobin (Hb) concentration in the supernatant of the mixture of trophozoites and HoRBCs after incubation for 1 h at 37°C. C and D. *E*. *histolytica* (Eh), *E*. *dispar* SAW1734RclAR cultured with *Crithidia fasciculata* (Ed) or *E*. *dispar* CYNO9:TPC (Cyno9) trophozoites (1×10^5^) were incubated with HoRBCs at 37°C for 1 h. C. Images of HoRBCs in a 96-well plate just after mixing with trophozoites (0 hr) and after incubation for 1 h with trophozoites (1 hr). D. Released hemoglobin (Hb) concentration in the supernatant of the mixture of trophozoites and HoRBCs after incubation for 1 h at 37°C. Data are the mean ± SD from 5 independent experiments. **p< 0.01, *p< 0.05 by ANOVA with Dunn test.

We have reported that *E*. *histolytica* trophozoites have hemolytic activity and that the activity can be blocked by an antibody recognizing M/C-Igl of EhIgl1 [12]. To assess whether *E*. *dispar* trophozoites also have this activity, the trophozoites were incubated with HoRBCs for 1 hr. The trophozoites of *E*. *dispar* SAW1734RclAR strain were cultured with *Pseudomonas aeruginosa* or with *Crithidia fasciculata* monoxenically because they were difficult to culture axenically. Trophozoites of another *E*. *dispar* strain, CYNO9:TPC, were able to be cultured axenically and the hemolytic activity of this strain was also assessed. As shown in Fig 4, SAW1734RclAR trophozoites cultured with *Pseudomonas aeruginosa* had hemolytic activity (Fig 4A and 4B) while trophozoites of the same strain cultured with *Crithidia fasciculata* and CYNO9:TPC strain did not have activity under the test conditions. The activity in SAW1734RclAR trophozoites cultured with *Pseudomonas aeruginosa* is due to contamination of hemolysin expressed in *Pseudomonas aeruginosa* [23].

### Hemolytic activity of Igl1 gene-silenced *Entamoeba histolytica*

Recombinant Igl proteins from *E*. *histolytica* and *E*. *dispar* showed similar hemolytic activities. However, expression of *Igl1* in trophozoites of *E*. *histolytica* is about twice as high as that in *E*. *dispar* trophozoites [8]. At the same time, *E*. *dispar* trophozoites did not have hemolytic activity (Fig 4). To evaluate whether the expression level of *Igl1* affects hemolytic activity, we generated *Igl1* gene-silenced (gs*Igl1*) *E*. *histolytica* strains and conducted the hemolytic assay.

**Fig 5:**
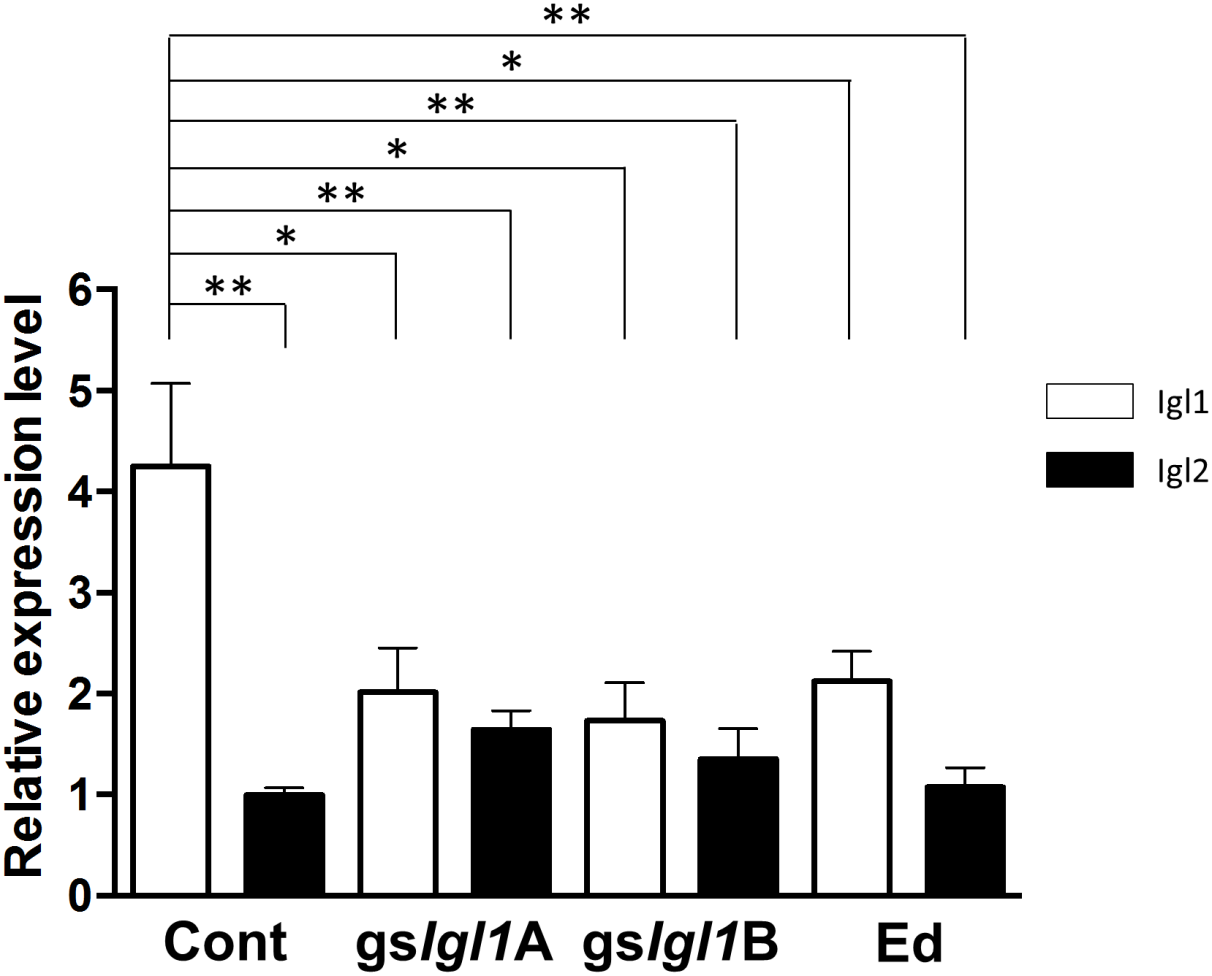
Real-time PCR analysis of *Igl* genes from *E*. *histolytica* and *E*. *dispar*. Expression levels of *Igl1* (open bars) and *Igl2* (filled bars) in trophozoites from *E*. *histolytica* strain with an empty vector (Cont), *E*. *histolytica* gs*Igl1* strains (gs*Igl1*A and gs*Igl1*B) and *E*. *dispar* SAW1734RclAR strain (Ed) were compared using *actin* as an internal standard. Expression levels are shown as values relative to the mean expression level of *Igl2* from Cont. Vertical bars indicate the S.E. of the mean from 3 experiments. *p< 0.05, **p< 0.01 by ANOVA with Dunn test.

Expression levels of *Igl1* and *Igl2* in the gene-silenced *E*. *histolytica* trophozoites were evaluated quantitatively by real-time PCR (Fig 5). *E*. *dispar* trophozoites expressed about a half the level of *Igl1* compared with *E*. *histolytica* trophozoites (Cont), as described previously [8]. Both *Igl1* gene-silenced *E*. *histolytica* strains (gs*Igl1*A and gs*Igl1*B) showed significantly lower expression of *Igl1*, but not *Igl2*, compared with vector control trophozoites (Fig 5). We could not generate an *Igl2* gene-silenced *E*. *histolytica* strain for unknown reason. Interestingly, *Igl2* expressions in *Igl1*-silenced *E*. *histolytica* strains were rather high compared with that in vector control trophozoites (Fig 5).

**Fig 6:**
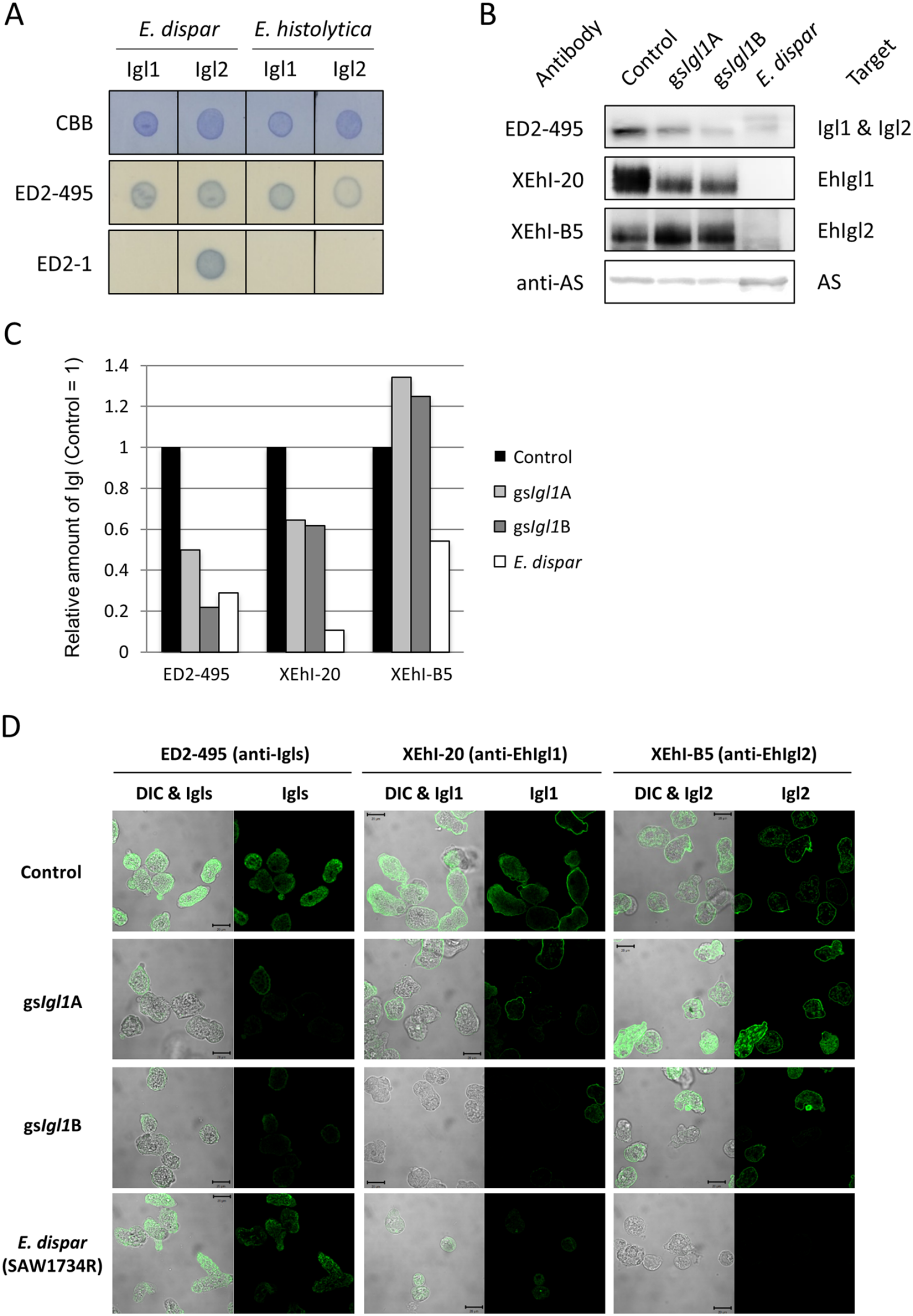
Establishment of Igl-attenuated *Entamoeba histolytica* trophozoites. A. Dot blot analysis of reactivity of ED2-495 against *E*. *histolytica* and *E*. *dispar* Igls. B. Western blot of Igls in control, gene-silenced *E*. *histolytica* and *E*. *dispar* trophozoites. C. Relative quantification of Igls expressed in control, gene-silenced *E*. *histolytica* and *E*. *dispar* trophozoites. D. Suppression of Igl1 protein expression by gene-silencing confirmed by IFA. Control: vector transfected *E*. *histolytica* trophozoite, gs*Igl1*: *Igl1* gene-silenced *E*. *histolytica* trophozoite, AS: ATP sulfurylase, DIC: differential interference contrast. Amino acid sequence alignment of ATP sulfurylase between *E*. *histolytica* and *E*. *dispar* is shown in S1 Fig.

Downregulation of Igl1 was further confirmed by Western blotting and cell staining (Fig 6). Western blotting by mAb ED2-495, which recognizes both Igl1 and Igl2 of *E*. *histolytica* and *E*. *dispar* (Fig 6A), showed 50–80% reduction of Igl expression in gs*Igl1*A and gs*Igl1*B strains, with this reduction due mainly to downregulation of Igl1 (Fig 6B and 6C). Expression levels of Igl2 in gs*Igl1*A and gs*Igl1*B strains were higher than in vector control trophozoites, in agreement with the real-time PCR results shown in Fig 5. Downregulation of Igl1 in gs*Igl1* strains was also confirmed using an immunofluorescence assay (Fig 6D). These results indicate that establishment of the gs*Igl1* strains was successful.

**Fig 7:**
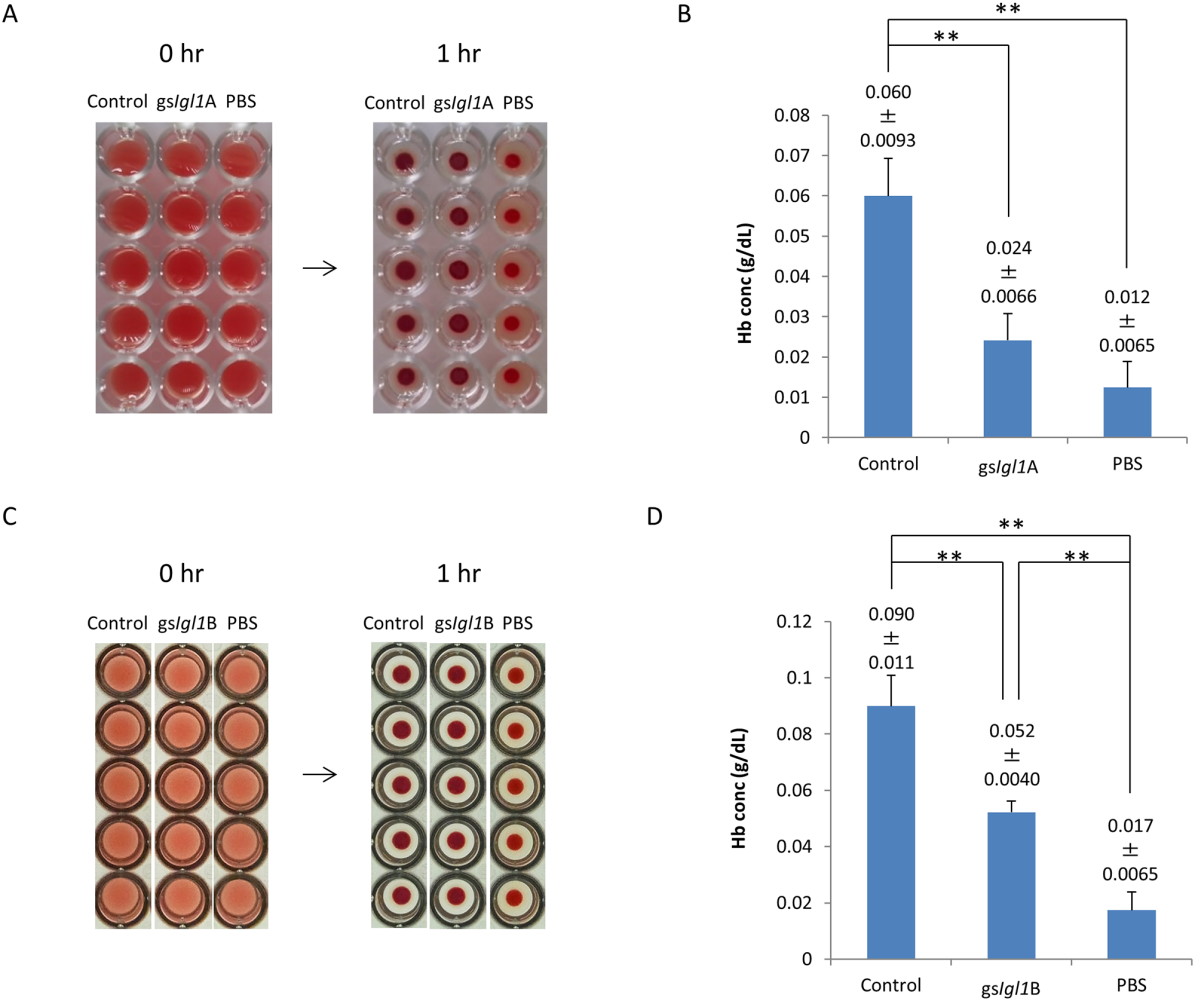
Hemolytic activities of Igl1-attenuated *Entamoeba histolytica* trophozoites. Vector transfected or *Igl1* gene-silenced *E*. *histolytica* trophozoites (1×10^5^) were incubated with HoRBCs at 37°C for 1 h. A and C. Images of HoRBCs in a 96-well plate just after mixing with trophozoites (0 hr) and after incubation for 1 h with trophozoites (1 hr). B and D. Released hemoglobin (Hb) concentration in the supernatant of a mixture of trophozoites and HoRBCs after incubation for 1 h at 37°C. Data are the mean ± SD from 5 independent experiments. **p< 0.01 by ANOVA with Dunn test. Control: vector transfected *E*. *histolytica* trophozoite, gs*Igl1*A and gs*Igl1*B: *Igl1* gene-silenced *E*. *histolytica* trophozoite.

After incubation of vector control or gs*Igl1*A trophozoites with HoRBCs for 1 h, the rim of the accumulated samples became vague in treatment with control trophozoites compared with gs*Igl1*A-treated samples (Fig 7A). For quantitative evaluation, supernatants of the incubated samples were collected and assayed for released Hb (Fig 7B). The hemolytic activity of gs*Igl1*A trophozoites (0.024±0.0066 g dL^-1^) was significantly lower than that of control trophozoites (0.060±0.0093 g dL^-1^). PBST treatment gave a [Hb] of 0.012±0.0065 g dL^-1^. *E*. *histolytica* gs*Igl1*B trophozoites also had a lower hemolytic activity than control trophozoites (Fig 7C and 7D), indicating that lower hemolytic activity reflects lower Igl1 expression in the parasites.

## Discussion

The *E*. *histolytica* lectin consists of three subunits, Hgl, Lgl and Igl, of which Hgl and Igl have lectin activities [24]. Recently, we found that EhIgl1 had hemolytic and cytotoxic activities [12]. Since *E*. *dispar*, a non-virulent species, also has an Igl1 subunit homologue, it is of interest to determine whether EdIgl1 has these activities. In this study, we showed that EdIgl1 has similar hemolytic activity to that of EhIgl1, with the site of this activity residing at the C-terminus in both proteins. There are two isoforms of Igl, and therefore, we also evaluated the hemolytic activities of EhIgl2 and EdIgl2. All EhIgls and EdIgls had hemolytic activity in our assay. This is the first study to show that *E*. *dispar* Igls and *E*. *histolytica* Igl2 lectins have hemolytic activities.

Factors related to the virulence of *Entamoeba* spp. remain unclear, despite several detailed studies [25–28]. Among the potential factors, both Hgl and Lgl lectin subunit expression are lower in *E*. *dispar* compared with *E*. *histolytica* [29]. Low expression of *Lgl1* was also found in an avirulent *E*. *histolytica* Rahman strain compared with the highly virulent *E*. *histolytica* HM-1:IMSS strain [30]. Expression of dominant negative Hgl or Lgl in the *E*. *histolytica* HM-1:IMSS strain and antisense inhibition of expression of Lgl in the same strain gave a less virulent strain [30–32]. Antisense inhibition of expression of EhCP5, an amoebic cysteine protease, in the HM-1: IMSS strain resulted in reduced virulence [33–35]. EhCP5 is missing in *E*. *dispar* [36] and is expressed at a lower level in the Rahman strain compared to the HM-1:IMSS strain [25]. Antisense inhibition of amoebapore expression in the HM-1:IMSS strain also decreases amoebic virulence [37]. Thus, many virulence-related molecules have been identified, but there may be additional factors related to amoebic virulence [38].

The Igl subunit also has vital roles in the pathogenicity of the parasite, including attachment to host cells and killing activities [12, 39, 40]. EhF-Igl1 and EhF-Igl2 have 83–84% amino acid sequence identity, while the amino acid sequence identity of EhF-Igl1 and EdF-Igl1 is 75–76% and that of EhF-Igl2 and EdF-Igl2 is 73–74% [8]. EhC-Igl1 and EdC-Igl1 have 76–77% amino acid sequence identity, with conserved cysteine residues. The Igls also have different expression levels in each species, with *Igl1* having higher expression in *E*. *histolytica* than in *E*. *dispar*, but *Igl2* having similar expression in the two species [8]. To assess whether the difference in level of Igl1 proteins affects the hemolytic activity of *E*. *histolytica*, we generated *Igl1* gene-silenced *E*. *histolytica* trophozoites and compared the activity with vector-transfected *E*. *histolytica* trophozoites. Interestingly, a 40% reduction of Igl1 protein expression led to a significant decrease in hemolytic activity.

This observation correlates with the weaker cytotoxicity of non-virulent *E*. *dispar* in a Gal/GalNAc lectin-mediated manner *in vitro* [41]. Erythrophagocytosis of *E*. *dispar* was observed in another study [42]. More importantly, *E*. *dispar* is pathogenic in experimental animals [43–46] and in humans [47, 48]. These effects have recently been reviewed [49]. The mechanism of the pathogenicity to *E*. *dispar* is still unclear, but it is possible that the incidence of infection by *Entamoeba* spp. is related to the expression levels of Igls and other related proteins. Further studies are needed to evaluate this possibility.

One of the interesting observations in this study was increased expression of Igl2 in both of *Igl1*-silenced strains. By contrast, decreased level of *Igl2* expression has been observed by short hairpin RNA-mediated knockdown of *Igl1* [50]. The discrepancy may be due to the difference of methods used to prepare transfectants.

## Supporting information

S1 Fig Amino acid sequence alignment of ATP sulfurylase between *E*. *histolytica* (XP_653570) and *E*. *dispar* (XP_001738584). Asterisks indicate the same amino acids.
(TIF)
