# Supplementary figures and images for: Comparison of hemolytic activity of the intermediate subunit of Entamoeba histolytica and Entamoeba dispar lectins
Source: PLoS One. 2017 Jul 27;12(7):e0181864. doi: 10.1371/journal.pone.0181864 (PMC5531476; doi:10.1371/journal.pone.0181864)

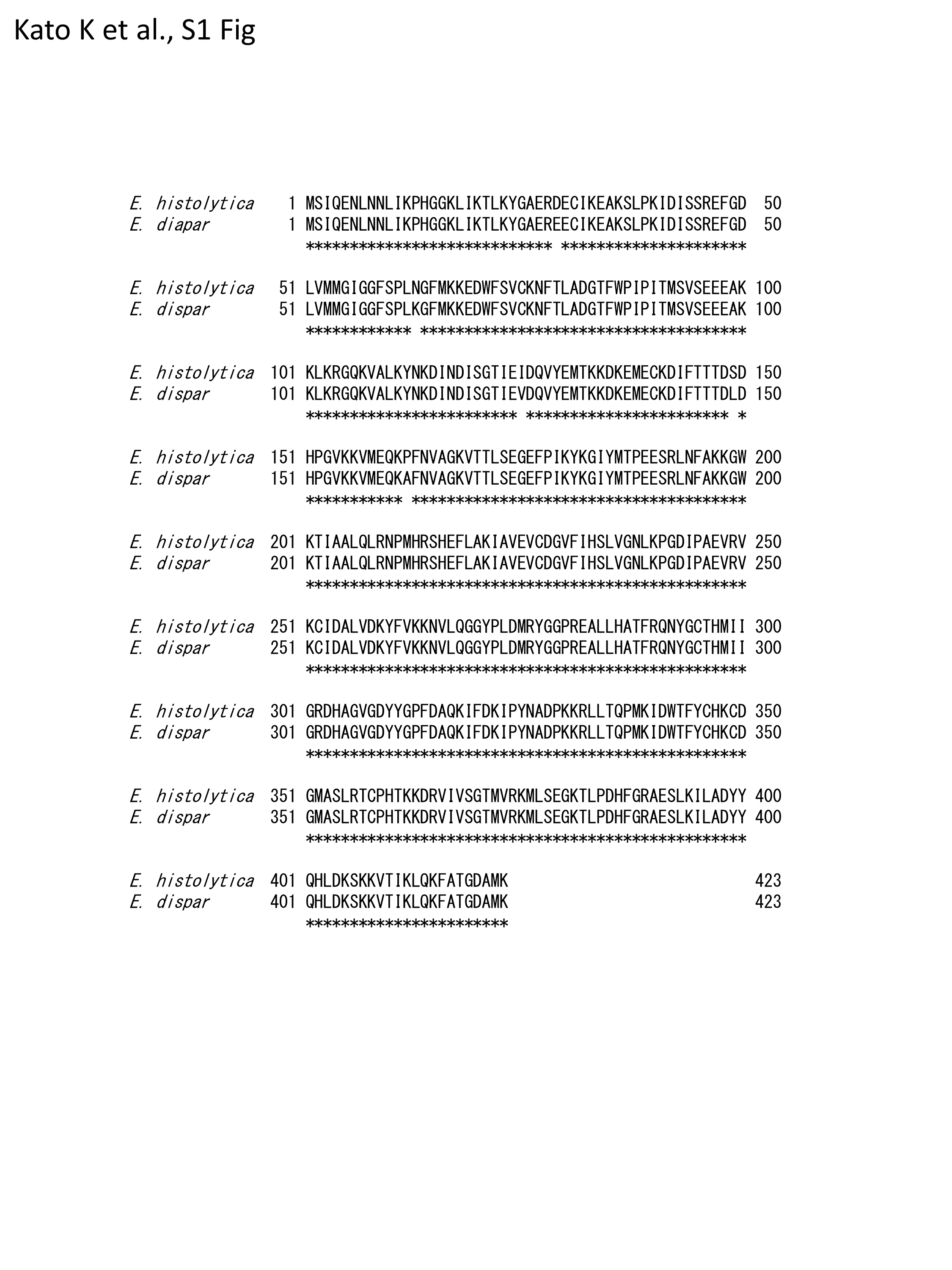

Supplement: S1 Fig — Asterisks indicate the same amino acids. (TIF) [file pone.0181864.s001.tif]
